# Unraveling the complex dynamics of signaling molecules in cellular signal transduction

**DOI:** 10.1093/pnasnexus/pgae020

**Published:** 2024-01-17

**Authors:** Shenqing Wang, Yi Zhang, Liangwei Zhang, Yan Huang, Jie Zhang, Kena Zhang, Yujie Huang, Gaoxing Su, Lingxin Chen, Bing Yan

**Affiliations:** Institute of Environmental Research at the Greater Bay Area, Key Laboratory for Water Quality and Conservation of the Pearl River Delta, Ministry of Education, Guangzhou University, Guangzhou 510006, China; Institute of Environmental Research at the Greater Bay Area, Key Laboratory for Water Quality and Conservation of the Pearl River Delta, Ministry of Education, Guangzhou University, Guangzhou 510006, China; CAS Key Laboratory of Coastal Environmental Processes and Ecological Remediation, Yantai Institute of Coastal Zone Research, Chinese Academy of Sciences, Yantai 264003, China; CAS Key Laboratory of Coastal Environmental Processes and Ecological Remediation, Yantai Institute of Coastal Zone Research, Chinese Academy of Sciences, Yantai 264003, China; School of Pharmacy, Binzhou Medical University, Yantai 264003, China; Institute of Environmental Research at the Greater Bay Area, Key Laboratory for Water Quality and Conservation of the Pearl River Delta, Ministry of Education, Guangzhou University, Guangzhou 510006, China; Institute of Environmental Research at the Greater Bay Area, Key Laboratory for Water Quality and Conservation of the Pearl River Delta, Ministry of Education, Guangzhou University, Guangzhou 510006, China; Institute of Environmental Research at the Greater Bay Area, Key Laboratory for Water Quality and Conservation of the Pearl River Delta, Ministry of Education, Guangzhou University, Guangzhou 510006, China; School of Pharmacy, Nantong University, Nantong, Jiangsu 226001, China; CAS Key Laboratory of Coastal Environmental Processes and Ecological Remediation, Yantai Institute of Coastal Zone Research, Chinese Academy of Sciences, Yantai 264003, China; Institute of Environmental Research at the Greater Bay Area, Key Laboratory for Water Quality and Conservation of the Pearl River Delta, Ministry of Education, Guangzhou University, Guangzhou 510006, China

**Keywords:** oxidative gradient, cell homeostasis, signaling, redox biology, polysulfides

## Abstract

Signaling molecules in cellular responses to foreign stimuli are described as static up- or down-concentration changes during signal transduction. This is because analytical methods for transducing molecules are much slower than the signaling events. In this study, we develop a dynamic cell model and reveal the temporal regulation of signal transduction events in response to reactive oxygen species (ROS). The model contained a set of 10 batches of redox-modified cells that mimic the temporal ROS accumulation events. Validating this dynamic cell model, we discover that cells survive early ROS attacks by activating the Nrf2/polysulfide/p62/CDK1 pathway. Nearly all signaling molecules exhibit time-dependent V-shape or inverse V-shape activation/feedback regulation dynamics in response to ROS accumulation. The results show that the dynamic cell model approach is invaluable for revealing complex signal intensity- and time-dependent cell signaling events.

**Significance Statement**

Our study presents a new cell model that tracks how cells regulate their internal signaling over time when exposed to external stimuli. This model uses cells modified to experience a buildup of cellular reactive oxygen species, a common challenge cells face. It reveals a survival strategy where cells activate a previously unrecognized pathway in response to this stress. A crucial discovery is the dynamic variations in the response intensities of all signaling molecules throughout the signaling process. This insight demonstrates the value of our model in understanding the complex and time-dependent ways cells communicate internally, an area previously poorly understood.

## Introduction

Multicellular organisms can respond dynamically to stimuli of varying intensity over time (1). Cells within these organisms also possess intricate feedback mechanisms to counteract the detrimental effects of excessive signaling (2). When a threshold is surpassed, the feedback systems (3, 4) can inhibit further activation or initiate counteractive cellular responses, rendering cellular signal transduction by each signaling molecule a nonlinear and dynamic process. In practice, the dominant method to quantify signaling molecules is western blotting. The method uses antibodies to quantify the static amounts of signaling proteins in the digested biological samples. The complex and prolonged processing procedures prevent this approach from presenting any time resolution (5) to capture fast molecular events, such as protein folding or conformational changes (6) during cell signaling. Similar issues also plague Southern blotting (7) or other similar methods characterizing signaling molecules. Therefore, in the existing literature, cells are reported to respond to stimuli in the static up-and-down arrows (8). Despite the identification of thousands of signaling molecules and hundreds of signaling pathways, the temporal cellular responses of the signaling molecules to stimulations are still unexplored. Therefore, developing cell models that accurately reflect cell responses to the temporal variation of stimuli holds the potential to advance our understanding of molecular signaling significantly.

Taking cellular reactions to reactive oxygen species (ROS) as an example, endogenous ROS, which is the main portion of cellular ROS, are generated in subcellular compartments and show a clear time-dependent concentration buildup within cells. Studying cellular responses to the accumulating ROS remains a technical challenge because cell models mimicking time-dependent intracellular ROS accumulation are unavailable. Traditionally, ROS is modeled by introducing external oxidants such as H_2_O_2_. Although different intracellular ROS concentrations in cells can be achieved by applying various doses of H_2_O_2_, this strategy has several shortcomings. First, external H_2_O_2_ induces only a ROS spike in seconds (9), distinct from the gradual generation and accumulation of endogenous ROS and cannot replicate this dynamic process. As a result, they only provide a static snapshot of disrupted molecular states and do not capture the authentic dynamics of cellular signaling (10). Second, because of their high reactivity, H_2_O_2_ forces cells to mobilize many stress-related mechanisms during cell penetration, distorting cellular responses, causing unintended oxidative shock, disrupting the redox signaling network, and even inducing cytotoxicity (10, 11). Therefore, a suitable dynamic cell model should mimic the time-dependent ROS accumulation, preferably having an intracellular ROS source that generates ROS mildly.

To counter this challenge, we designed and synthesized 10 gold nanoparticles (GNPs) with different redox potencies taking advantage of the high engineerability of nanoparticles (12, 13). Introducing these GNPs into cells helped create a set of 10 cells with a stepwise increase in cellular ROS levels that simulate the time-dependent ROS accumulation. These cells allow us to investigate temporal biological consequences in response to the time-dependent buildup of intracellular ROS. Using our dynamic cell model, we discovered that below the oxidative stress threshold, cells mitigate early ROS assaults by activating the Nrf2/polysulfide/p62/CDK1 pathway to induce cell cycle arrest at the G2/M phase. We revealed that most signaling molecules exhibited time-dependent V-shape or reversed V-shape dynamics in response to ROS accumulation. These results advanced our understanding of how signaling molecules function in a time-dependent fashion when cells respond to external stimuli.

## Results

### Redox-modified cells mimic temporal ROS accumulation inside cells

**Fig. 1.**
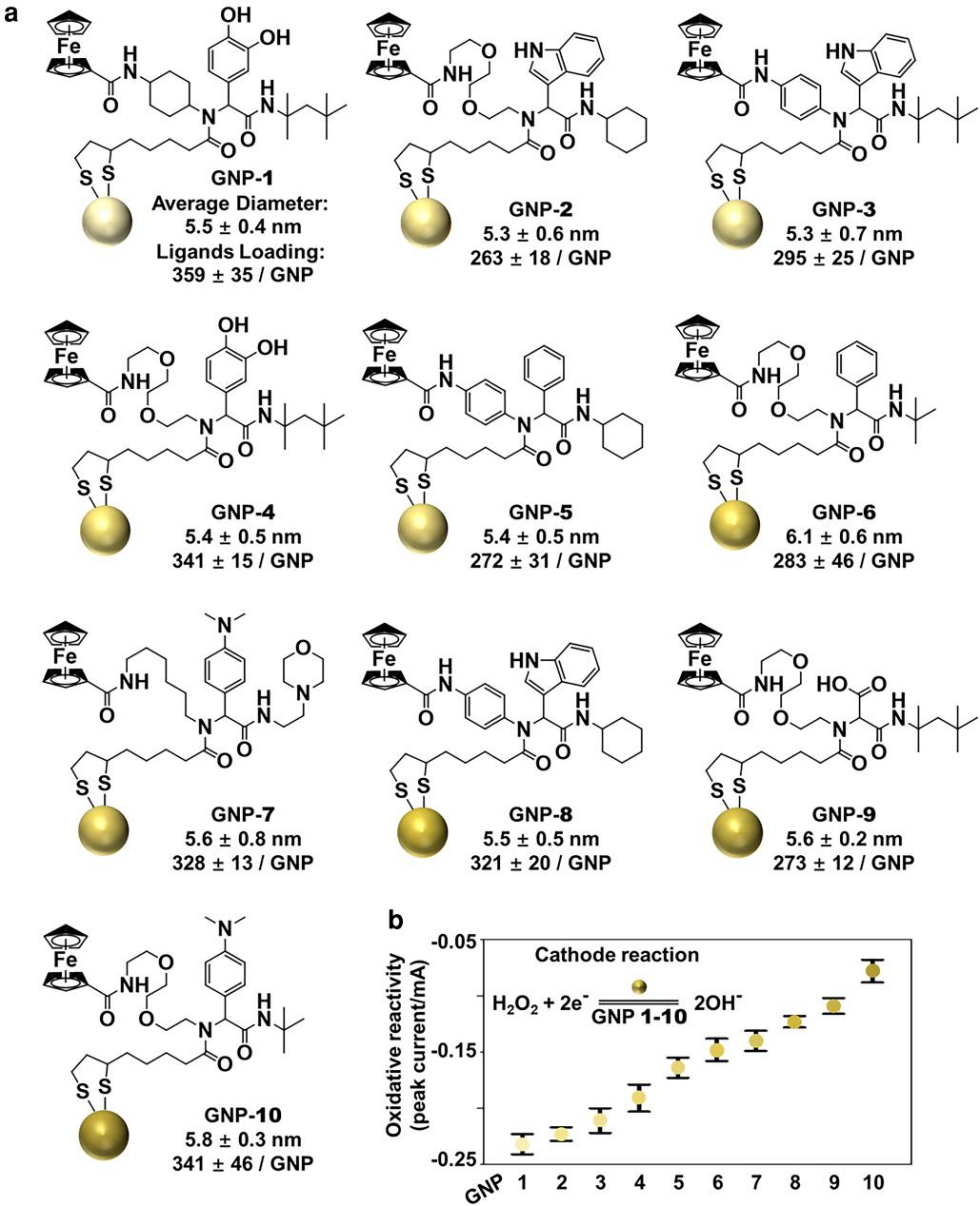
Ten engineered GNPs with a stepwise increase of redox reactivities. a) GNPs 1–10 were synthesized by reducing HAuCl_4_ with sodium borohydride and modified with the indicated ligands. The average diameters (average values from analyses of at least 70 GNPs determined by TEM) and the incorporated ligands per GNP (determined by the quantitative analysis of Au/Fe content using ICP-MS) are shown (for details, see Materials and methods). The gradually deepened color in GNPs indicates an increase in their oxidative reactivity. b) The redox potencies of 10 GNPs were determined using cyclic voltammetry measurements using the H_2_O_2_ oxidation as the cathode reaction.

To create sequential batches of cells (1–10) with progressively increasing ROS levels, we incubated cells 1–10 with 50 μg/mL of GNPs 1–10 (Fig. 1a), respectively, for 24 h. Because GNPs 1–10 have been engineered, each with a specific ferrocenyl-containing ligand (Fig. 1a) on their surface to have progressively higher oxidative reactivities as determined by cyclic voltammetry (Figs. 1b and S1).

**Fig. 2.**
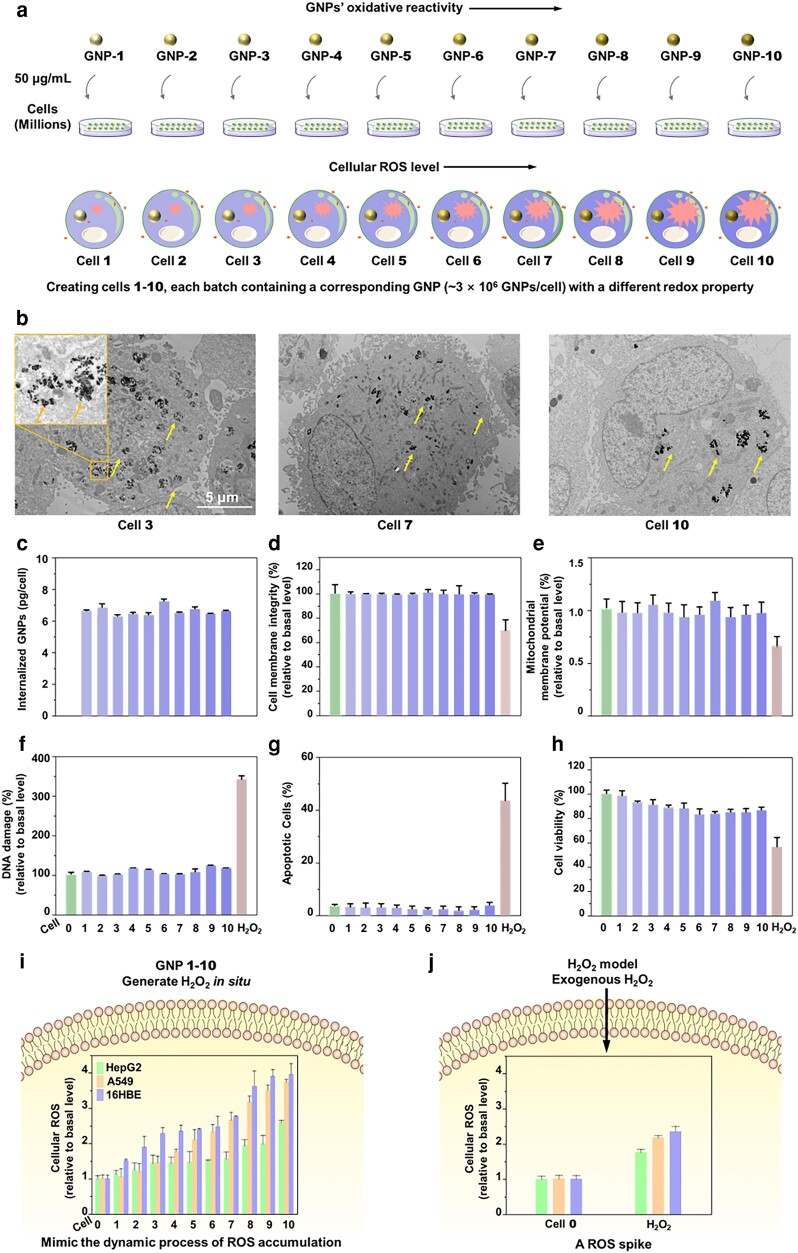
Generation of cells with progressively higher ROS levels. a) The schematic shows 10 sequential batches of cells incubated with GNPs 1–10 (50 μg/mL), respectively, for 24 h to form 10 redox-modified cells with progressively higher cellular ROS. b) TEM images show cell uptakes of GNPs in A549 cells after 24 h. c) ICP-MS analysis of lysed cells indicates an average of 3.0 ± 0.3 × 10^6^ GNPs/cell in cells 1–10. The gradually deepened column color indicates an increase in cellular ROS. d) LDH leakage test shows GNP uptake did not affect cell membrane integrity except for H_2_O_2_ (200 μM). e) The mitochondrial membrane potentials of GNP-treated cells are similar to cell 0, characterized by JC-1 staining, except for H_2_O_2_-treated cells. f) Quantitative analysis using a DNA damage marker 8-hydroxyguanine shows minimal DNA damage in cells 1–10 except for H_2_O_2_-treated cells. g) Annexin V/PI double staining revealed no cell apoptosis in cells 1–10 except for H_2_O_2_-treated cells. h) Cell viability determined by the CellTiter-Lumi luminescence method indicates that >80% of cells remained viable after GNP incorporation in cells 1–10 except for H_2_O_2_-treated cells. Our dynamic ROS cell model i) is compared with the conventional H_2_O_2_ spike model j). DCFH-DA fluorescent probe was used to determine cellular ROS level. Data in c)–i) are from three independent experiments and error bars represent one SD.

Cells 1–10 that respectively contain GNPs 1–10 possess progressively higher ROS levels (Fig. 2a) without oxidative stress damage (Fig. 2). Transmission electron microscopy (TEM) images showed that GNPs were internalized after 24 h (Fig. 2b). Inductive coupling plasma mass spectrometry (ICP-MS) measurements of lysed cells quantified that each cell for cells 1–10 internalized an average of 3.0 ± 0.3 × 10^6^ GNPs (Fig. 2c). Importantly, the nontoxic dose of GNPs (50 µg/mL) did not affect cell membrane integrity (Fig. 2d) or mitochondrial membrane potential (Fig. 2e) nor trigger DNA damage (Fig. 2f), cell apoptosis (Fig. 2g), or cytotoxicity (>80% viable, Fig. 2h). Cellular ROS levels in cells 1–10 increased from 0.81 to 2.84 times the basal ROS level (Fig. 2i). Moreover, the cellular ROS level of cells 1–10 is positively correlated with the oxidative activity of GNPs 1–10 (Fig. S2). Cell 10, with the highest ROS level, also did not display any notable cytotoxicity, indicating that ROS concentration up to 2.84 times the cell basal level is within the homeostatic range. In comparison, exogenous oxidants such as H_2_O_2_ (200 μM, a commonly used dose), which induced only 77% of the cellular ROS level in cell 10, compromised cell membranes, reduced mitochondrial membrane potential, and induced DNA damage, cell apoptosis, and cytotoxicity (Fig. 2d–h). Together, these results show that cells 1–10 generated a cellular ROS gradient in situ without causing oxidative stress damage. This stepwise increase in ROS across the 10 redox-modified cells forms a dynamic cell model to simulate the temporal process of ROS initiation and accumulation inside cells. It enables time-dependent cell signaling events in response to ROS to be investigated.

Because the maintenance of ROS homeostasis varies across cell types and their dysplasia status (14), we further examined whether redox-modified cells with an oxidative gradient can be universally achieved with other cell types. Following the procedure for A549 cells, we incorporated GNPs 1–10 into the human liver cancer cell line HepG2 and normal bronchial epithelial cell line 16HBE cells. Like A549 cells, redox-modified HepG2 and 16HBE cells were >80% viable (Fig. S3) and generated ROS gradients (Fig. 2i). Moreover, cell behavior also reflected the unique characteristics of each specific cell line. For example, at the same GNP concentration, HepG2 cells internalized on average 32 and 44% more GNPs per cell (Fig. S3) but displayed 29 and 40% lower ROS gradients than A549 and 16HBE cells, respectively (Fig. 2i). This is likely because HepG2 cells, which detoxify, inherently generate more GSH (glutathione) antioxidants than A549 and 16HBE cells. Comparing cell 10 from all three redox-modified cell lines, HepG2 cells displayed 127 and 79% higher intracellular GSH/GSSG (oxidized glutathione) ratios than A549 and 16HBE cells, respectively (Fig. 2j). These results show oxidative gradients can be established in multiple cell types, allowing temporal and molecular signaling events under different physiological and disease conditions to be studied in detail.

### Nrf2 regulates dynamic changes in GSH and polysulfides in response to ROS

**Fig. 3.**
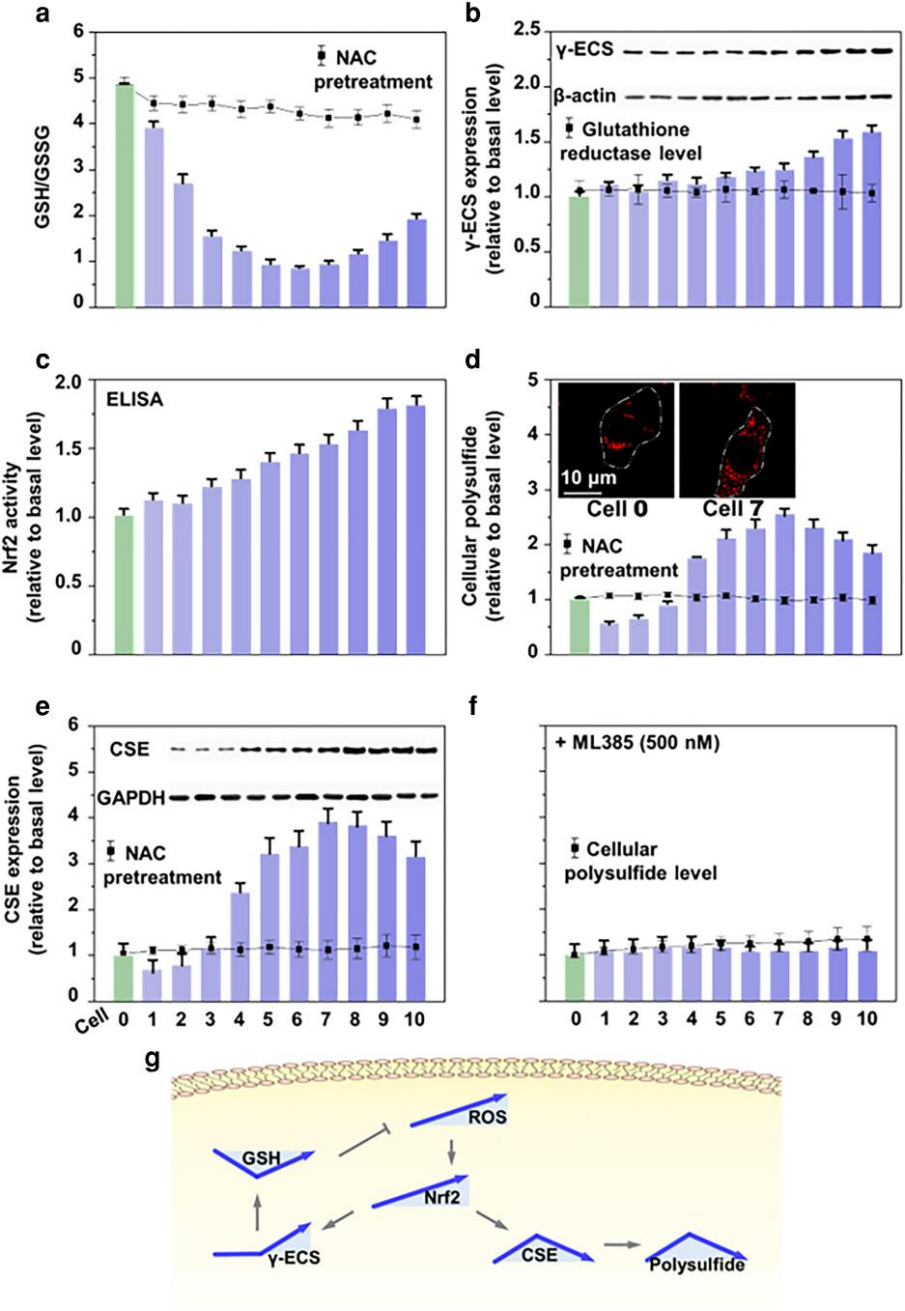
ROS-induced V-shape and reversed V-shape changes in GSH and polysulfide. a) GSH/GSSH ratio in redox-modified A549 cells 1–10 displays a V-shape trend in response to increasing cellular ROS. The gradually deepened column color indicates an increase in cellular ROS. b) Western blot (inset) and DTNB colorimetric analysis show that ROS increase up-regulates γ-ECS but not glutathione reductase expression. c) ELISA analysis reveals ROS enhances Nrf2 activity. d) As determined by a fluorescent probe, Hcy-Mito, polysulfide levels display a reversed V-shape pattern as ROS accumulates. The inset shows images of cells 0 and 7 in which cell outlines are shown with white dotted lines. e) Western blot analysis (inset) indicates that CSE expression presents a reverse V-shape trend as ROS increases. f) Inhibiting Nrf2 activity with ML385 suppresses all changes in CSE and polysulfide. g) In response to the dynamic accumulation of cellular ROS, a progressive increase of Nrf2 expression activates a V-shape change in GSH and a reversed V-shape change in polysulfide. All experimental values were from three independent experiments. Error bars represent one SD in bar charts and two in dot-line plots.

To validate this dynamic ROS cell model, we first examined the temporal regulation of GSH, an early responder to cellular ROS. Analyzing the GSH/GSSG ratio variation across the 10 redox-modified A549 cells, we found a V-shape changing trend with an inflection point near cells 6 and 7 (Fig. 3a). As ROS increased linearly, the GSH/GSSG ratio gradually decreased to about 0.17-fold of the basal level from cells 1 to 6 and then increased to 0.20-, 0.23-, 0.30-, and 0.39-fold of the basal level from cells 7 to 10. Western blot analysis showed constant glutathione reductase (the enzyme responsible for reducing GSSG to GSH) throughout, and the up-regulated γ-glutamylcysteine synthetase (γ-ECS; the rate-limiting enzyme for GSH synthesis) expression in cells 7–10 (Figs. 3b and S4). This suggested that the dynamic changes in the GSH/GSSG ratio were due to increased GSH biosynthesis rather than rapid GSSG reduction. Further, the loss of the V-shape trend when cellular ROS is inhibited with *N*-acetyl-l-cysteine (NAC) indicated that this dynamic V-shape change was caused by the linear increase in cellular ROS and the cellular biochemical regulations.

We next found a linearly increasing pattern (Fig. 3c) of Nrf2, a key antioxidant regulator in response to cellular ROS (15). Because ROS is known to trigger polysulfide biosynthesis (16), we also quantified the changes in cellular polysulfides in response to increasing ROS using a cell-based fluorescent probe, Hcy-Mito (17) (Figs. 3d and S5). Polysulfide levels showed a reversed V-shaped pattern with an inflection point at cell 7. This pattern was mirrored by the expression of cystathionine γ-lyase (CSE), a rate-limiting enzyme for polysulfide biosynthesis (Figs. 3e and S4). NAC treatment erased all changes in intracellular CSE and polysulfides. Meanwhile, inhibition of GSH synthesis using γ-ECS inhibitor, buthionine sulfoximine (BSO), resulted in a mild increase in polysulfide synthesis, likely to compensate for the loss of cellular GSH (Fig. S6) but did not impact polysulfides’ response to ROS. While GSH’s antioxidative role is well-established, the role of polysulfide in redox signaling is unknown. Previously, it was reported that Nrf2 up-regulates GSH biosynthesis in response to ROS (18). We found that inhibiting Nrf2 with ML385 abolished the reversed V-shape changes in CSE and polysulfides (Fig. 3f). Together, these findings demonstrate that intracellular ROS independently triggers dynamic changes in CSE expression (or polysulfide synthesis) and GSH synthesis, and Nrf2 has a dual regulatory role in modulating GSH and polysulfide levels in response to ROS (Fig. 3g).

### Nrf2 regulates V-shape temporal changes in p62 through polysulfide

**Fig. 4.**
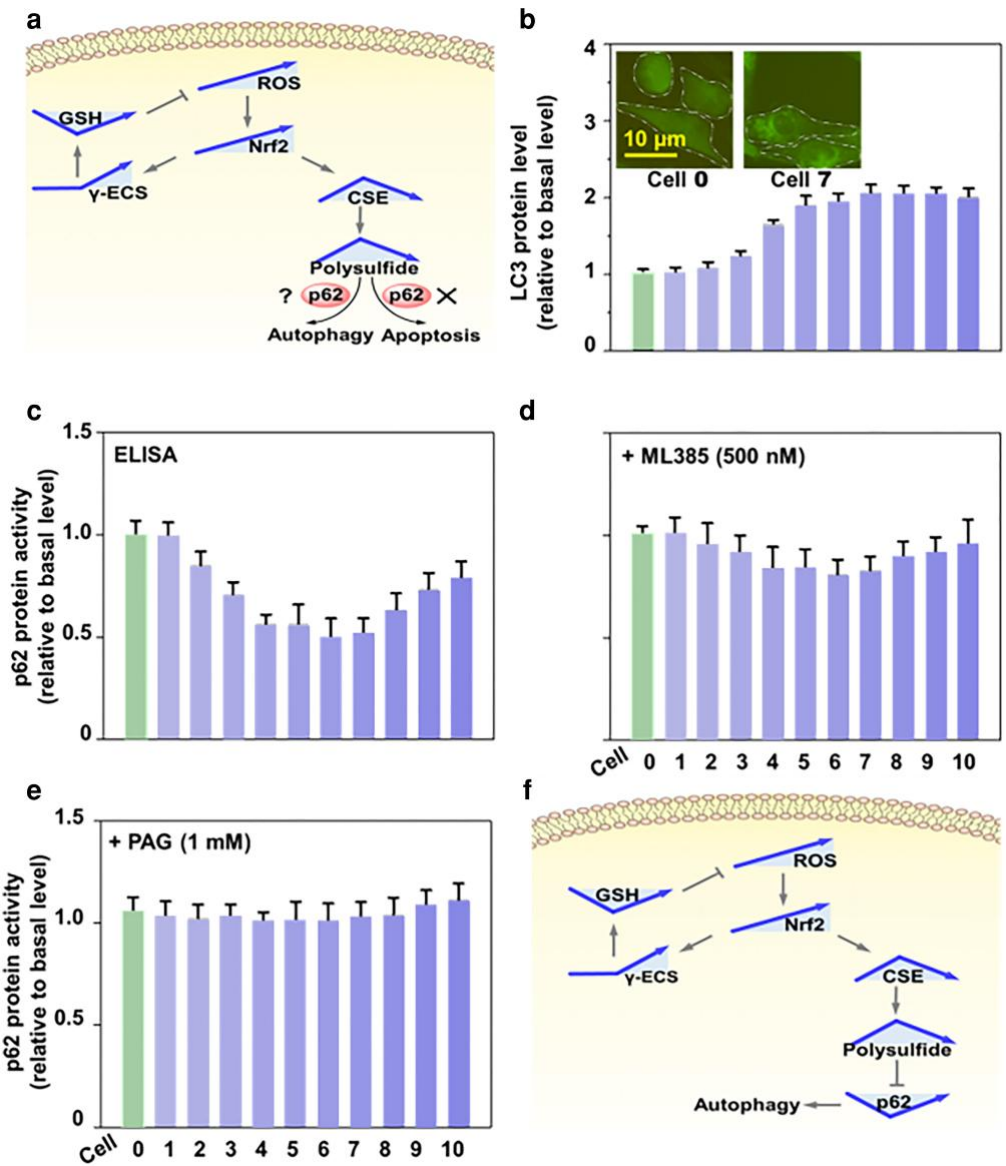
Nrf2 regulates a V-shape changing pattern in p62 through polysulfides. a) Nrf2-induced dynamic changes in polysulfide do not induce cell apoptosis (Fig. 2g), but whether it influences p62 or triggers cell autophagy is unknown. b) Increasing autophagosomes and the level of autophagy protein marker LC3, in response to cellular ROS indicate cell autophagy. Inset: Fluorescence images of LC3-GFP-U87 cells 0 and 7 with fluorescently-labeled autophagosomes. c) p62 protein activity responds to cellular ROS following a V-shape pattern. d) Inhibiting polysulfide with PAG (1 mM) or e) Nrf2 with ML385 (500 nM) eliminates ROS-induced changes in p62. f) Nrf2 modulates a V-shape p62 expression and promotes cell autophagy through a reversed V-shape change in polysulfide. All experimental values were from three independent experiments. Error bars represent one SD.

Autophagy, a cell survival mechanism activated by ROS, works in concert with apoptosis (19). In mammals, the Keap1-Nrf2 pathway and autophagy constitute critical cellular defense systems that counteract oxidative damage and uphold homeostasis (20). Linking both systems together is the ubiquitin-binding autophagy receptor protein, p62/SQSTM1 (21). Given that the ROS range produced in cells 1–10 did not trigger cell apoptosis (Fig. 2g), we sought to examine whether autophagy was induced and if so, whether p62 (22) was involved (Fig. 4a). Both western blot analysis of autophagy marker, LC3, and fluorescent imaging of autophagosomes in LC3-green fluorescent protein (GFP)-U87 cells we developed previously (23) showed that GNPs 1–10 activated cell autophagy (Figs. 4b and S7). However, protein analysis revealed that Nrf2 suppressed p62 in response to low levels of ROS in cells 1–6 but initiated enhancement of p62 at higher ROS levels from cell 7 onwards, forming a V-shape trend (Fig. 4c). The p62 enhancement in response to elevated ROS is consistent with a previous report (24). However, the early suppression of p62 is a novel finding achieved with this dynamic ROS cell model.

Using chemical biology techniques to investigate the relationship between p62 and its upstream regulators, we discovered that blocking either Nrf2 activity with ML385 (Fig. 4d) or polysulfide synthesis with CSE inhibitor, propargylglycine (PAG, Fig. 4e) eased p62 changes in the cells. These findings suggest that polysulfides, under the control of Nrf2, act upstream of p62, thus negatively regulating p62 expression at lower levels of cellular ROS (Fig. 4f).

### ROS induces V-shape changes in CDK1 through polysulfides and p62

**Fig. 5.**
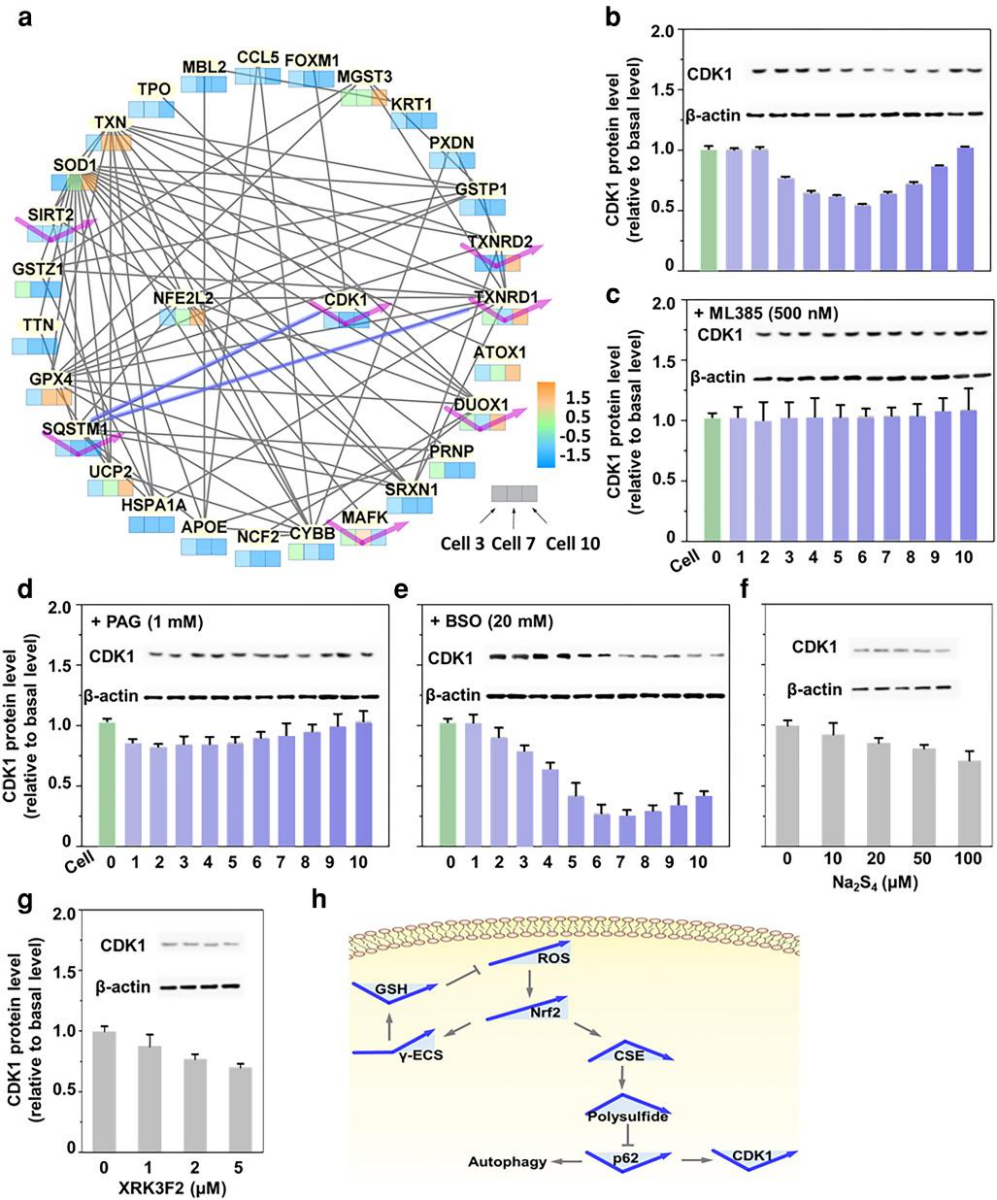
V-shape changes in CDK1 mediated by polysulfides and similar p62. a) Protein–protein interaction network of 29 antioxidant genes regulated by ROS increase in cells 3, 7, and 10 is obtained from PCR array analysis of 80 antioxidant-related genes. Genes were imported into STRING for interaction analysis and visualized using Cytoscape. Three-bar indicators show gene expression levels in cells 3, 7, and 10; V-shape changed genes are highlighted. b) Western blot analysis shows CDK1 expression in redox-modified A549 cells 1–10 displays a V-shape trend. (c) Inhibiting Nrf2 with ML385 eliminates CDK1 activity. d) Inhibiting polysulfide with PAG eliminates CDK1 activation. e) Inhibiting GSH synthesis with BSO has no effect and even slightly enhances CDK1. CDK1 level is suppressed by external polysulfide precursor Na_2_S_4_ (f) and p62 inhibitor XRK3F2 (g) in a dose-dependent manner. h) Schematic model showing the dynamic regulation of cellular CDK1 through the Nrf2/polysulfide/p62 pathway. All experimental values were from three independent experiments. Error bars represent one SD.

p62 fulfills its prosurvival or proapoptotic role by engaging with diverse factors (25). To identify the interaction partners for p62 in cells responding to ROS, we analyzed the differential gene expression profiles of cells 3, 7, and 10 relative to cell 0 using an antioxidant mechanism PCR array containing 80 related genes. Results showed that ROS-regulated 29 genes (Fig. 5a). Among ROS-altered genes, 7 showed a monotonic decrease, and 15 showed a monotonic increase. Only seven genes (TXNRD2, SQSTM1, SIRT2, CDK1, TXNRD1, DUOX1, and MAFK) displayed a V-shape changing pattern (Table S1). Examining the association of these genes using the STRING database and visualizing the results with Cytoscape (version 3.7.1), we found that p62 (SQSTM1) modulates a set of genes, including CDK1 and TXNRD1 that also displayed a V-shape change. TXNRD1, known as thioredoxin reductase 1, is a crucial antioxidant enzyme that maintains cellular redox homeostasis. CDK1, cyclin-dependent kinase 1, is a protein critical in regulating cell cycle regulation in response to cellular ROS (26). CDK1 plays a central role in mitosis and is an essential CDK in cell cycle regulation. The activity of CDK1 is dynamically regulated to ensure timely mitotic processes.

Western blot analysis revealed that CDK1 levels declined in cells 1–6 but recovered in cells 7–10 (Fig. 5b). This pattern aligns with the V-shape changes observed for p62 (Fig. 4c). Loss of the V-shape change in CDK1 upon inhibition of Nrf2 activity (Fig. 5c) and polysulfide biosynthesis (Fig. 5d) confirms that Nrf2 modulates CDK1 expression via polysulfides. Consistent with the association between polysulfides and CDK1, inhibiting GSH biosynthesis with BSO partially enhanced the biosynthesis of polysulfides to compensate for the loss of GSH (Fig. S6) and bolstered polysulfide's inhibitory effects on CDK1 expression (Fig. 5e). To further prove the causal relationship between polysulfides and CDK1, we treated the cells with an exogenous polysulfide precursor, Na_2_S_4_, and measured CDK1 expression. The dose-dependent suppression of CDK1 expression further demonstrates the regulatory role of polysulfides (Fig. 5f). Treating cells with a p62 inhibitor, XRK3F2, suppressed CDK1 levels in a dose-dependent fashion (Fig. 5g). These findings suggest that polysulfides suppress CDK1 expression in the early phases of ROS accumulation below the oxidative stress. This function is mediated through p62 (Fig. 5h).

### Polysulfides temporally regulate cell cycle arrest via p62

**Fig. 6.**
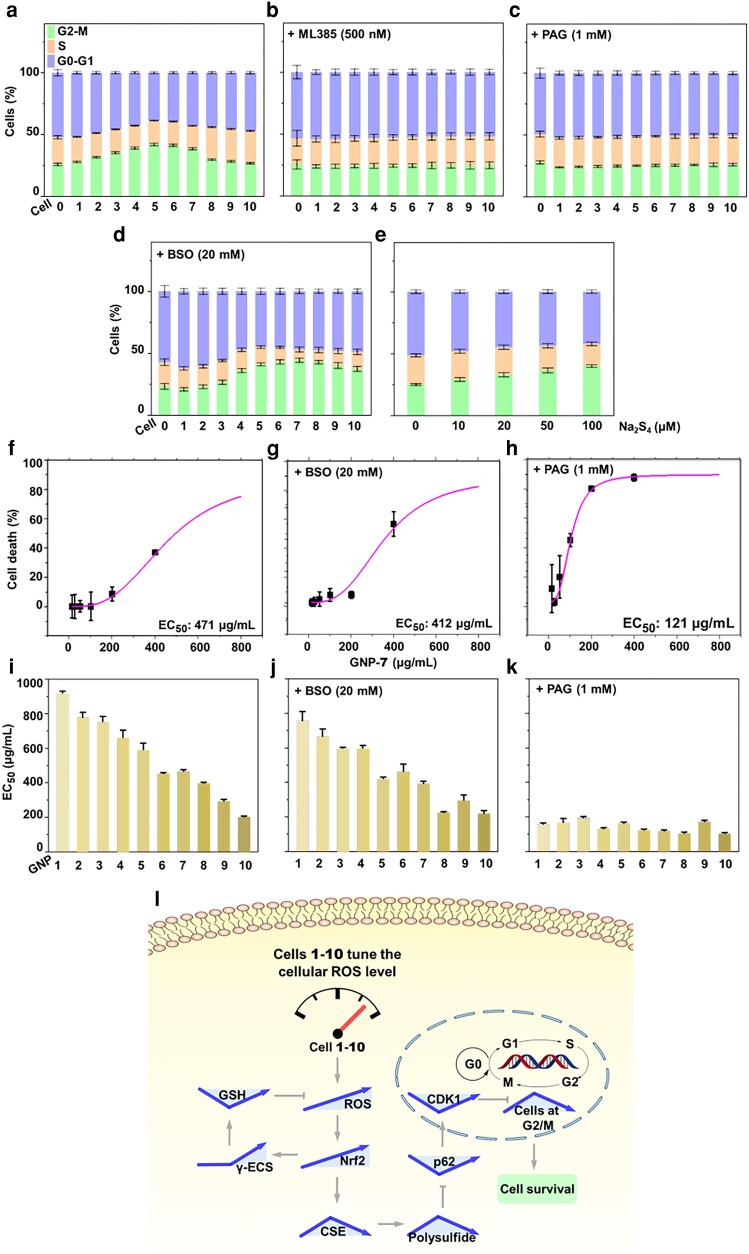
Dynamic induction of cell cycle arrest by low-level ROS facilitates cell survival. a) Cell cycle arrest at the G2/M phase in redox-modified A549 cells 1–10 displays a reversed V-shape, as determined using propidium iodide staining. Inhibiting Nrf2 activity with ML385 (b) or polysulfide synthesis with PAG (c) eliminates time-dependent cell cycle arrest, while inhibiting GSH with BSO (d) does not. e) Polysulfide precursor Na_2_S_4_ enhances cell cycle arrest in a dose-dependent manner. f–h) EC_50_ value of cells treated with GNP 7 (471 μg/mL) does not change significantly in the presence of BSO (412 μg/mL) but decreases significantly in the presence of PAG (121 μg/mL). i–k) EC_50_ values of cells treated with GNPs 1–10 without inhibitor (i) with BSO (j) or PAG (k) show cell death is inevitable when polysulfide is inhibited, and Nrf2-regulated polysulfide promotes cell survival. All experimental values were from three independent experiments. Error bars in (f–h) represent two SDs. All other error bars represent one SD. l) ROS-triggered dynamic signal transduction pathways induce cell survival. As cellular ROS accumulates, a gradual increase in Nrf2 triggers dynamic V-shape changes in GSH and reversed V-shape changes in polysulfides. Polysulfides suppress both p62 and CDK1 in a time-dependent V-shape pattern. These dynamic changes facilitate cell survival by arresting the cell cycle at the G2/M phase in a time-dependent reversed V-shape pattern.

Because CDK1 is a crucial kinase for advancing through the G2/M checkpoint, its dynamic suppression by ROS could obstruct cell cycle progression. Cell cycle analysis revealed that compared with cells at the basal ROS level (cell 0, 25% of cells in the G2/M phase), the cell cycle was arrested at the G2/M phase. For cells 1–10, this arrest temporally displayed a reversed V-shape pattern (Fig. 6a). This dynamic alteration in cell cycle arrest at the G2/M phase was abolished when Nrf2 function (Fig. 6b) or polysulfide biosynthesis (Fig. 6c) was inhibited. GSH inhibition, which induced a compensatory polysulfide biosynthesis, did not significantly impact this trend (Fig. 6d). Conversely, supplementing polysulfides with a precursor, Na_2_S_4_, enhanced G2/M arrest in a dose-dependent fashion (Fig. 6e). These results indicate that the cell cycle is temporarily arrested at the G2/M phase in a reversed V-shape by the dynamic changes in signaling molecules in the signal transduction cascade of the Nrf2/polysulfide/p62/CDK1 pathway.

Under stressful conditions, halting cell proliferation by arresting the cell cycle at the G2/M phase is critical because it allows damaged DNA to be repaired and prevents any damaged DNA from propagating to daughter cells. We hypothesized that the CDK1-induced G2/M arrest might be essential for the survival of cells impacted by ROS. We evaluated the half-maximal effective concentration (EC50) values of GNPs 1–10 in A549 cells to investigate this hypothesis. With GNP 7, for instance, we found that inhibiting GSH biosynthesis with BSO did not significantly alter the EC_50_ value (471 μg/mL before inhibition vs. 412 μg/mL after inhibition; Figs. 6f, g and S8). Inhibiting polysulfides with PAG, however, drastically decreased the EC_50_ value from 471 to 121 μg/mL, showing a significant increase in cytotoxicity (Fig. 6h). For GNPs 1–10 with progressively higher oxidative reactivities, the decreasing EC_50_ values from 915 to 198 μg/mL show heightened cytotoxicity (Fig. 6i). GSH inhibition with BSO did not change this trend (Fig. 6j), but polysulfide inhibition caused the EC_50_ values to fall dramatically to an average of 143 ± 9 μg/mL across GNPs 1–10 (Fig. 6k). Together, these results demonstrate that ROS-induced intracellular polysulfides, rather than GSH, shielded cells from death through the signaling pathway of Nrf2/polysulfide/p62/CDK1 (Fig. 6l).

## Discussion

Human cells have developed flexible signaling processes to defend against foreign attacks. However, the mechanisms underlying these processes remain elusive. Cell models serve as crucial tools for comprehending cell signaling pathways. Existing cell models solely focus on measuring steady-state concentrations of signaling molecules, thus overlooking the dynamic nature of actual molecular signaling events. To address this gap, we develop a dynamic cell model in this study, which offers unprecedented insights into the time-dependent responses of cells.

This investigation revealed the dynamic changes in a cascade of signaling molecules in response to ROS. To develop a dynamic ROS cell model, we utilized GNPs with distinct redox reactivities to induce controllable amounts of ROS inside cells. This approach created 10 batches of redox-modified cells, each displaying specific levels of cellular ROS depending on the redox activities of corresponding GNPs. The gradual elevation of cellular ROS across these 10 batches of cells established an oxidative gradient that faithfully emulates the time-dependent initiation and accumulation of endogenous ROS. Therefore, our dynamic cell model monitors the temporal biological consequences of ROS-triggered signaling processes below the oxidative stress threshold by capturing continuous snapshots of time-dependent cellular events. This innovative approach enables a comprehensive understanding of the dynamic nature of cellular responses to ROS.

For example, GSH depleted first and then rose as ROS accumulated, constituting a V-shape dynamic response. This distinctive V-shape progression was also extended to other signaling molecules during signal transduction, such as V-shape changes for p62 and CDK1 and reversed V-shape patterns for CSE, polysulfides, and G2/M-phase cell accumulation. These dynamic responses in every signaling molecule would have remained concealed using conventional ROS spike models that solely measure the final steady-state concentration. By highlighting the dynamics of molecular signaling events, our findings underscore the intricate nature of intracellular activation and feedback regulation within ROS-induced signaling networks, surpassing previous understandings limited to simple up-and-down arrows commonly found in molecular signaling literature.

This study also unveiled a remarkable survival strategy employed by cells, as they halted cell cycle progression at the G2/M phase in response to early ROS attacks. Under these conditions, polysulfides were crucial in regulating p62 and CDK1 to induce cell cycle arrest. This novel discovery would have eluded traditional ROS spike models, as they tend to cause unrelated cellular perturbations that hinder such insights. Unveiling this missing link pathway not only highlights the value of our cell model in exploring cellular responses during the initial ROS assault but also emphasizes the importance of the early activation of cellular damage surveillance and repair mechanisms.

We also demonstrate that GNPs with diverse redox properties can establish ROS gradients in different cell types. This innovative approach enables the investigation of temporal molecular events that reflect cellular signal transduction dynamics with unprecedented detail under various physiological and pathological conditions. For example, this methodology holds promise for exploring the reversible dynamics of neurodegenerative processes (27) and designing targeted cancer therapies (28) that capitalize on the vulnerable time points when antioxidant activities in cancer cells are at their weakest. Furthermore, general cell signaling processes may possess similar dynamic characteristics. Therefore, by embracing the principles of this approach, researchers can establish similar dynamic cell models to unravel the dynamic nature of cellular signaling events beyond the scope of ROS-induced pathways.

## Materials and methods

### Reagents and cell lines

All chemical reagents used in this study were purchased from Sigma-Aldrich (St Louis, MO, USA) except otherwise indicated. CSE (30068) antibody was purchased from Cell Signaling Technology (Boston, MA, USA). β-Actin (AM1021b) and GAPDH (AM1020b), and Cdc2 (CDK1) (AP1497b) antibodies were purchased from Abgent (San Diego, CA, USA). Cell lysis buffer (R0278) and protease/phosphatase inhibitor cocktail (P8340) were from Millipore (Burlington, MA, USA). Dulbecco's modified Eagle medium (DMEM) of high glucose, RPMI1640, and minimum essential medium (MEM) were purchased from Thermo Scientific (Waltham, MA, USA). Fetal bovine serum (FBS) was from Sigma-Aldrich. Human phospho-SQSTM1/p62-Ser349 and SQSTM1/p62 ELISA kits were from MSKBIO. Antioxidant mechanisms PCR array was purchased from WcGene Biotech. The RNeasy mini kit and GSH/GSSG assay kit were purchased from Sigma-Aldrich and Beyotime Biotech.

Cell lines A549, HepG2, and 16HBE were purchased from the American Type Culture Collection. U87-GFP-LC3 cell line expressing LC3-GFP fusion protein was constructed by introducing retroviral construct MSCV-IRES-LC3-GFP into U87 cells.

### Synthesis and characterization of GNPs 1–10

GNPs 1–10 were synthesized by the sodium borohydride reduction method. Briefly, ligands were synthesized using a Ugi multicomponent reaction by condensing carboxylic acid, amide, aldehyde, and isonitrile in one step in methyl alcohol solution. Ten GNPs were obtained by reducing chloroauric acid with sodium borohydride in DMF/H_2_O solution at room temperature with the presence of different ligands. After the reaction, unreacted ligands and DMF were removed by washing with PBS. The final products were stored in high-purity water.

GNP concentration was measured using ICP-MS (Agilent 7900, Agilent Technologies, Inc., Santa Clara, CA, USA). Particle morphology and size were determined using a JEM-1011 JEOL TEM (JEOL, Tokyo, Japan). Surface ligand density was assessed by measuring Au:Fe mass ratio using ICP-MS. Dynamic hydrodynamic diameters and zeta potentials in water (50 μg/mL) were metered using a Malvern Nano Z Zetasizer (Malvern Instruments Inc., Malvern, UK). The partition coefficient was determined using a modified “shaking flask” method. Hydrophobicity was scaled by log_10_ (partition coefficient) (log *P*) value, which was calculated as the log_10_ (distribution ratio of GNPs in *n*-octanol to water).

Hydrogen peroxide reduction by GNPs was determined by cyclic voltammetry on a CHI 660A electrochemical workstation (Chenhua Instruments, Shanghai, China). Glassy carbon and silver/silver chloride electrodes were selected as working and reference electrodes. GNPs of the same surface area (5 × 10^16^ nm^2^/mL, 10 μL) were added onto the surface of electrodes. Cyclic voltammetry was recorded in 0.01 M PBS (prior to ultrasonic deaeration) with 10 mM H_2_O_2_. The potential was scanned from −1.40 to 0.60 V, with an initial potential of 0.00 V and a scanning rate of 0.02 V/s.

### Cell culture and cytotoxicity assay

All cell cultures were conducted in 5% CO_2_ at 37 °C. A549, HepG2, and 16HBE cells were cultured in RPMI-1640, DMEM, and MEM media. All media were supplemented with 10% (V/V) FBS, 100 U/mL penicillin, and 100 μg/mL streptomycin. Cells in the logarithmic growth phase were used for GNP exposure and toxicity assessment. Cells were seeded into a 96-well plate with 6k cells/well in 100 μL medium for the cytotoxicity test. After overnight culture, cells were treated with 0, 12.5, 25, 50, 100, 200, and 400 μg/mL GNPs for 48 h. Cell viability was then measured using CellTiter-Glo Assay on a VICTOR X2 Multilabel Plate Reader (PerkinElmer Inc., Waltham, MA, USA). When inhibitors were used, cells were pretreated with inhibitors at appropriate concentrations for 2 h before GNP exposure for 24 h. Inhibitors used in this study include NAC (ROS inhibitor, 5 mM), PAG (CSE inhibitor, 1 mM), BSO (γ-ECS inhibitor, 20 mM), or ML385 (Nrf2 inhibitor, 500 nM).

### GNP cell uptake

After GNP exposure, cell number was counted before preparing lysate using aqua regia. The intracellular element Au amount was measured using ICP-MS (Agilent 7900, Agilent Technologies, Inc.).

### Intracellular ROS measurement

Cells in 24-well plates (60k cells/well) were treated with 50 μg/mL GNPs or 200 μM H_2_O_2_ for 24 h. After being washed with PBS, cells were incubated with 2,7-dichloro-dihydro-fluorescein diacetate (DCFH-DA, 10 μM) for 30 min in the darkness. After three washes with PBS, fluorescence intensity was detected on a VICTOR X2 Multilabel Plate Reader.

### GSH/GSSG measurement

Cells in 12-well plates (60k cells/well) were detached with trypsin and underwent frozen/thawed cycles twice in liquid nitrogen and 37 °C water bath before total glutathione measurement using GSH and GSSG Assay Kit. Thirty microliters of protein removal reagent were added to each tube and mixed well by the vortex. After that, samples underwent frozen/thawed cycle twice in liquid nitrogen and a 37 °C water bath. Samples were incubated at 4 °C for 5 min, followed by a centrifuge at 10,000 × *g* at 4 °C for 10 min. A supernatant was used for total glutathione measurement. for the kinetic catalysis experiment, the absorbance of 5,5′-dithiobis-(2-nitrobenzoic acid) (DTNB) at 412 nm (A_412_) was measured on VICTOR X2 Multilabel Plate Reader (PerkinElmer Inc., Waltham, MA, USA) every 3 min for 30 min.

### LDH leakage test

After treatment in 96-well plates (60k cells/well), take 80 μL of the supernatant and add it to the corresponding well of a new 96-well plate, and add 60 μL of LDH detection working solution to each well. Mix well and incubate at room temperature in the dark for 30 min. Then measure the absorbance at 490 nm. Calculate by subtracting the absorbance of the background blank control well from the measured absorbance of each group.

### Mitochondrial membrane potentials

After treatment in 12-well plates (60k cells/well), A549 cells were detached using trypsin. Add 0.5 mL of JC-1 staining solution and mix well. Incubate at 37 °C in a cell incubator for 20 min. Centrifuge 600 × *g* at 4 °C for 4 min, precipitate cells, and discard supernatant. Add 1 mL of JC-1 staining buffer to resuspend cells, centrifuge 600 × *g* at 4 °C for 4 min, precipitate cells, and discard the supernatant. After resuspension with 120 μL JC-1 staining buffer, detect with a fluorescence enzyme-linked immunosorbent assay. When detecting JC-1 monomer, set the excitation wavelength to 490 nm and the emission wavelength to 530 nm; when detecting JC-1 polymer, set the excitation wavelength to 525 nm and the emission wavelength to 590 nm.

### Cellular polysulfides measurement

Cells in confocal dishes (60k cells/dish) were incubated with fluorescent probe Hcy-Mito (1 μg/mL) for 30 min. Image acquisition was performed using an OLYMPUS FV2000 laser scanning confocal microscope, with the excitation and acquisition wavelength set as 633 and 700–800 nm, respectively. Quantitative analysis of fluorescence intensity was performed using ImageJ.

### Cell cycle, apoptosis, and autophagy analysis

Cells were fixed in cold 70% ethanol overnight at −20 °C for cell cycle analysis using Guava flow cytometry. For apoptosis analysis, live cells were incubated with Nexin-V reagent at room temperature for 25 min. Cells treated with H_2_O_2_ (200 μM) for 2 h were used as a positive control. All cytometry results were analyzed using ModFit.

For cell autophagy analysis, U87-GFP-LC3 cells treated with GNPs for 24 h were washed with PBS three times before imaging by an inverted fluorescence microscope. Autophagy was quantified by counting GFP-LC3 puncta. Three biological repeats were set up for each experiment.

### Gene interaction analysis

RNA was prepared using the *RNeasy Mini Kit*. Gene expression analysis was performed using Antioxidant Mechanisms PCR Array according to the manufacturer's instructions. After the sample was extracted, the OD (optical density) value was measured by Quickdrop to quantify the RNA concentration. Initial denaturation: 95 °C, 10 min. Denaturation: 95 °C, 15 s; annealing: 60 °C, 60 s; 50 cycles. The ΔCt of genes related to each pathway in each treatment group was calculated. The difference in corresponding gene expression between the treatment group and the control group was calculated by 2^−ΔCt^. Differential expression cutoff values were set up at ≤0.5 or ≥1.5. Gene interaction was analyzed by searching the STRING protein interaction database and visualized using Cytoscape (version 3.7.1).

### Statistical analysis

Origin 2015 was used for statistical analyses. One-way ANOVA followed by least-significant difference was performed to determine significance. Differences were considered significant when *P* < 0.05. Numerical data are presented as the mean ± SD (X ± SD).

## Supplementary Material

pgae020_Supplementary_Data

## Data Availability

All data are included in the manuscript and Supplementary material.

## References

[1] Zhang Z , Costa M. 2021. p62 functions as a signal hub in metal carcinogenesis. Semin Cancer Biol. 76:267–278. 33894381 10.1016/j.semcancer.2021.04.014 PMC9161642

[2] Aman A , Nguyen M, Piotrowski T. 2010. Wnt/β-catenin signaling regulates the morphogenesis of the zebrafish lateral line. Dev Biol. 344:433.

[3] Liu Y , Shepherd EG, Nelin LD. 2007. MAPK phosphatases–regulating the immune response. Nat Rev Immunol. 7:202–212. 17318231 10.1038/nri2035

[4] Canovas B , Nebreda AR. 2021. Diversity and versatility of p38 kinase signalling in health and disease. Nat Rev Mol Cell Biol. 22:346–366. 33504982 10.1038/s41580-020-00322-w PMC7838852

[5] Mohseni AH , Taghinezhad-S S, Casolaro V, Lv Z, Li D. 2023. Potential links between the microbiota and T cell immunity determine the tumor cell fate. Cell Death Dis. 14:154. 36828830 10.1038/s41419-023-05560-2 PMC9958015

[6] Cohen L , Walt DR. 2018. Highly sensitive and multiplexed protein measurements. Chem Rev. 119:293–321. 30152694 10.1021/acs.chemrev.8b00257

[7] Hanash S , Taguchi A. 2010. The grand challenge to decipher the cancer proteome. Nat Rev Cancer. 10:652–660. 20733593 10.1038/nrc2918

[8] Mu Q , Du G, Chen T, Zhang B, Yan B. 2009. Suppression of human bone morphogenetic protein signaling by carboxylated single-walled carbon nanotubes. ACS Nano. 3:1139–1144. 19402638 10.1021/nn900252j

[9] Bienert GP , Schjoerring JK, Jahn TP. 2006. Membrane transport of hydrogen peroxide. Biochim Biophys Acta. 1758:994–1003. 16566894 10.1016/j.bbamem.2006.02.015

[10] Janssen-Heininger YMW , et al 2008. Redox-based regulation of signal transduction: principles, pitfalls, and promises. Free Radic Biol Med. 45:1–17. 18423411 10.1016/j.freeradbiomed.2008.03.011 PMC2453533

[11] Gruszczyk AV , et al 2022. Mitochondrial metabolism and bioenergetic function in an anoxic isolated adult mouse cardiomyocyte model of in vivo cardiac ischemia-reperfusion injury. Redox Biol. 54:102368. 35749842 10.1016/j.redox.2022.102368 PMC9234472

[12] Mu Q , et al 2014. Chemical basis of interactions between engineered nanoparticles and biological systems. Chem Rev. 114:7740–7781. 24927254 10.1021/cr400295a PMC4578874

[13] Bai X , et al 2020. Regulation of cell uptake and cytotoxicity by nanoparticle core under the controlled shape, size, and surface chemistries. ACS Nano. 14:289–302. 31869202 10.1021/acsnano.9b04407

[14] Henderson JR , Fulton DA, McNeil CJ, Manning P. 2009. The development and in vitro characterisation of an intracellular nanosensor responsive to reactive oxygen species. Biosens Bioelectron. 24:3608–3614. 19553099 10.1016/j.bios.2009.05.029

[15] Moi P , Chan K, Asunis I, Cao A, Kan YW. 1994. Isolation of NF-E2-related factor 2 (Nrf2), a NF-E2-like basic leucine zipper transcriptional activator that binds to the tandem NF-E2/AP1 repeat of the beta-globin locus control region. Proc Natl Acad Sci USA. 91:9926–9930. 7937919 10.1073/pnas.91.21.9926 PMC44930

[16] Zhang X , Zhang L, Gao M, Wang Y, Chen L. 2020. A near-infrared fluorescent probe for observing thionitrous acid-mediated hydrogen polysulfides formation and fluctuation in cells and in vivo under hypoxia stress. J Hazard Mater. 396:122673. 32361129 10.1016/j.jhazmat.2020.122673

[17] Huang Y , Yu F, Wang J, Chen L. 2016. Near-infrared fluorescence probe for in situ detection of superoxide anion and hydrogen polysulfides in mitochondrial oxidative stress. Anal Chem. 88:4122–4129. 26926943 10.1021/acs.analchem.6b00458

[18] Moinova HR , Mulcahy RT. 1999. Up-regulation of human γ-glutamylcysteine synthetase regulatory subunit gene involves binding of Nrf2 to an electrophile response element. Biochem Biophys Res Commun. 261:661–668. 10441483 10.1006/bbrc.1999.1109

[19] Chen X , Clark J, Guan JL, Kumar AR, Zheng Y. 2015. Susceptibility of AML to chloroquine therapy is independent of autophagy. Blood. 126(23):1262.

[20] Hayes JD , Dinkova-Kostova AT. 2014. The Nrf2 regulatory network provides an interface between redox and intermediary metabolism. Trends Biochem Sci. 39:199–218. 24647116 10.1016/j.tibs.2014.02.002

[21] Zhang W , Feng C, Jiang H. 2020. Novel target for treating Alzheimer's diseases: crosstalk between the Nrf2 pathway and autophagy. Ageing Res Rev. 65:101207. 33144123 10.1016/j.arr.2020.101207

[22] Komatsu M , et al 2010. The selective autophagy substrate p62 activates the stress responsive transcription factor Nrf2 through inactivation of Keap1. Nat Cell Biol. 12:213–223. 20173742 10.1038/ncb2021

[23] Wu L , et al 2014. Correction to tuning cell autophagy by diversifying carbon nanotube surface chemistry. ACS Nano. 8:5366. 10.1021/nn500376w PMC5586106 24552177

[24] Ran W , et al 2019. Activation of Keap1/Nrf2/p62 signaling alleviates high phosphate-induced calcification of vascular smooth muscle cells by suppressing reactive oxygen species production. Sci Rep. 9:10366. 31316111 10.1038/s41598-019-46824-2 PMC6637199

[25] Moscat J , Diaz-Meco MT. 2009. p62 at the crossroads of autophagy, apoptosis, and cancer. Cell. 137:1001–1004. 19524504 10.1016/j.cell.2009.05.023 PMC3971861

[26] Chen Q , et al 2012. Amplified in breast cancer 1 enhances human cholangiocarcinoma growth and chemoresistance by simultaneous activation of Akt and Nrf2 pathways. Hepatology. 55:1820–1829. 22213475 10.1002/hep.25549

[27] Sarkar A , Rasheed MSU, Singh MP. 2023. Redox modulation of mitochondrial proteins in the neurotoxicant models of Parkinson's disease. Antioxid Redox Signal. 38:824–852. 36401516 10.1089/ars.2022.0106

[28] Xu R , et al 2018. Extracellular vesicles in cancer-implications for future improvements in cancer care. Nat Rev Clin Oncol. 15:617–638. 29795272 10.1038/s41571-018-0036-9

