# Supplementary material for: Unraveling the complex dynamics of signaling molecules in cellular signal transduction
Source: PNAS Nexus. 2024 Jan 17;3(1):pgae020. doi: 10.1093/pnasnexus/pgae020 (PMC10810328; doi:10.1093/pnasnexus/pgae020)
Supplement: pgae020_Supplementary_Data [file pgae020_supplementary_data.docx]

Supporting Information

for

**Unraveling the Complex Dynamics of Signaling Molecules in Cellular Signal Transduction**

Supplementary section

Figure S1. Characterization of GNPs **1**-**10**.

Figure S2. The positive correlation between the cellular ROS level of cells **1-10** and the oxidative activity of GNPs **1-10**.

Figure S3. GNPs **1-10** exhibit similar effects in multiple cell types.

Figure S4. Western blot characterizes the protein expression.

Figure S5. Polysulfide production in A549 cells as imaged by confocal microscope.

Figure S6. The interaction between GSH and polysulfides.

Figure S7. The confocal microscope image of U87 cells reflects autophagy.

Figure S8. Cytotoxicity of GNPs **1-10**.

Table S1. Summary of differentially expressed genes screened.


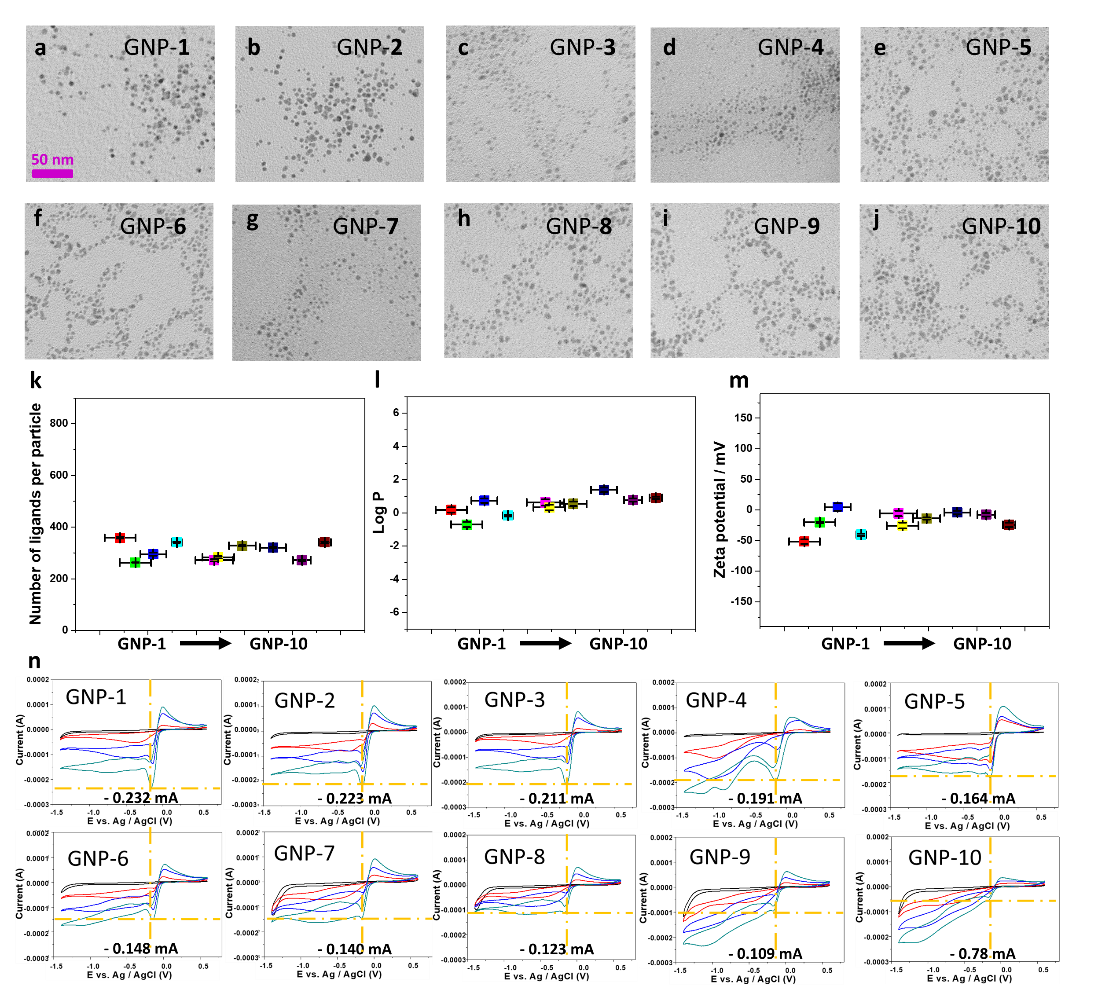


Figure S1. Characterization of GNPs **1**-**10**. (a-j) Transmission electron microscopy (TEM) images of GNPs **1-10**. TEM images of the GNPs were taken using a JEOL-1011 transmission electron microscope (JEM, Tokyo, Japan) at 100 KV. The images were acquired using an AMT 2k CCD camera. Image data were analyzed by ImageJ. (k) Surface ligand density shown as number of ligand molecules per particle. (l) Hydrophobicity of GNPs **1-10** shown as Log P. Log P is Log_10_ (distribution ratio of GNPs in n-octanol phase and water phase). (m) Zeta potential of GNPs **1-10**. (n) Cyclic voltammetry of GNPs **1-10**. Cyclic voltammetry was recorded in 0.01 M PBS (prior ultrasonic deaeration, black curve) or in PBS with H_2_O_2_ in concentrations of 0.3% (red curve), 0.6% (blue curve), 0.9% (green curve). Peak current at -0.20 V (vs. Ag/AgCl) of each GNPs (orange dotted line) showed gradient changes in redox activity.


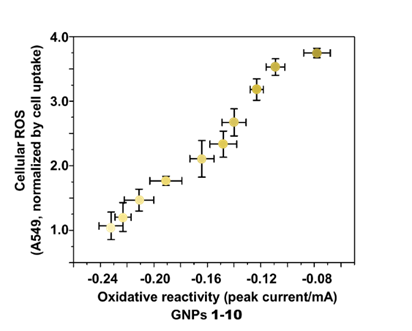


Figure S2. The positive correlation between the cellular ROS level of cells **1-10** and the oxidative activity of GNPs **1-10**. The cellular ROS levels of cells **1-10** have been normalized by cellular uptake to reflect the redox activity of GNPs **1-10**, while excluding the influence of uptake.


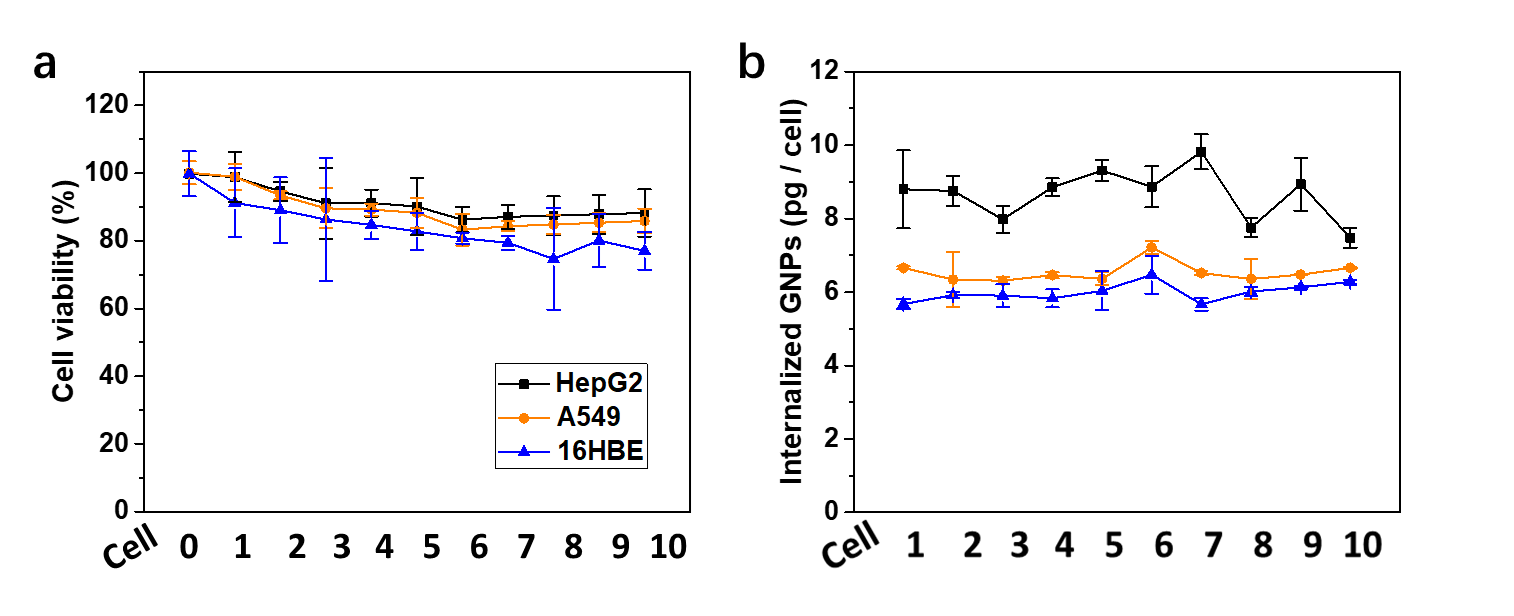


Figure S3. GNPs **1-10** exhibit similar effects in multiple cell types. (a) HepG2, A549, and 16HBE cells incubated with GNPs **1-10** (50 μg/mL) for 24 hours to form redox-modified cells **1-10** maintained > 80% viability. (b) Cell uptakes of GNPs **1-10** by three cell lines are consistent.


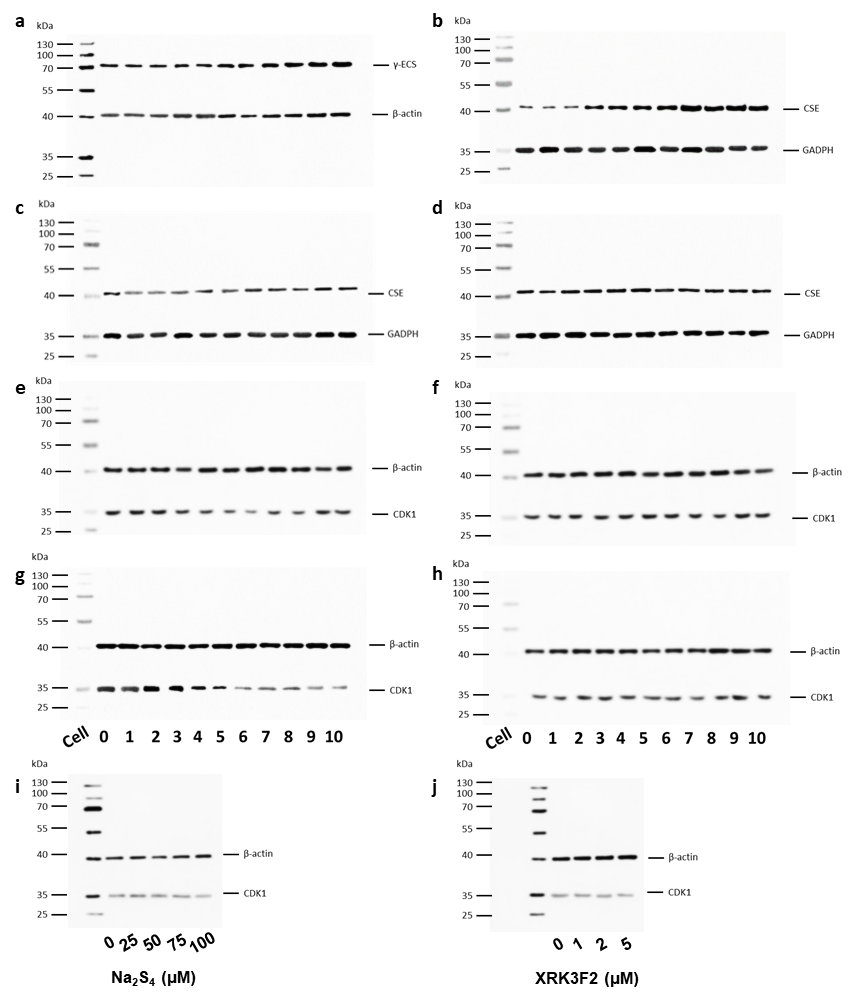


Figure S4. Western blot characterizes the protein expression level of cells treated with GNPs. (a) γ-ECS, treat cells with GNPs for 24 hours; (b) CSE, treat cells with GNPs for 24 hours; (c) CSE, cells were pretreated with NAC for 2 hours, and then treated with GNP for 24 hours; (d) CSE, pretreated cells with ML385 for 2 hours, followed by treatment with GNP for 24 hours; (e) CDK1, treated cells with GNP for 24 hours; (f) CDK1, pretreated cells with ML385 for 2 hours, followed by treatment with GNP for 24 hours; (g) CDK1, pretreated cells with BSO for 2 hours, followed by treatment with GNP for 24 hours; (h) CDK1, pretreated cells with PAG for 2 hours, then treated with GNP for 24 hours; (i) CDK1, treated cells with Na_2_S_4_ for 24 hours; (j) CDK1, treated cells with XRK3F2 for 24 hours. After protein sample extraction, quantitative analysis was performed using the BCA method, 20 μg protein was used for western blot assay. The band intensity was quantiﬁed using ImageJ.


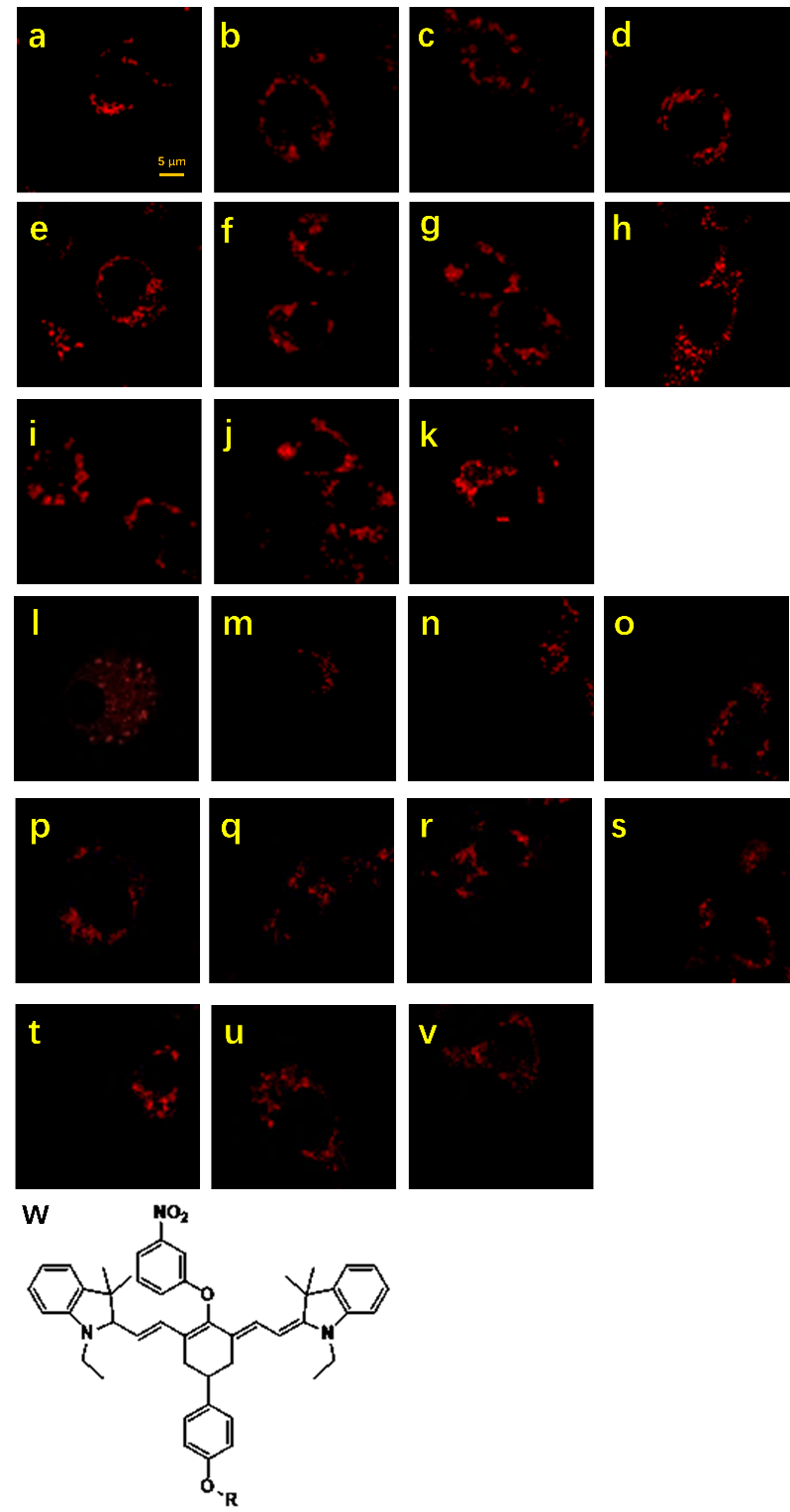


Figure S5. Polysulfide production in A549 cells as imaged by confocal microscope. Before imaged, cells were treated with GNPs (50 μg/mL) for 24 hours, followed by incubation with Hcy-Mito for 30 min. (a-k) Images of Cells **0-10**. (l-v) Images of Cells **0-10** pretreated with NAC. (w) Structure of polysulfide-specific fluorescent probe Hcy-Mito (1), a Cyanine7 derivative with a maximum absorption at 770 nm (ε_770 nm_ = 8.9 × 10^4^ M^−1^ cm^−1^). Image acquisition was performed using OLYMPUS FV2000 laser scanning confocal microscope (Olympus Corporation, Tokyo, Japan), with the excitation and acquisition wavelength set as 633 nm and 700-800 nm, respectively. The quantitative analysis of fluorescence intensity was performed using ImageJ.


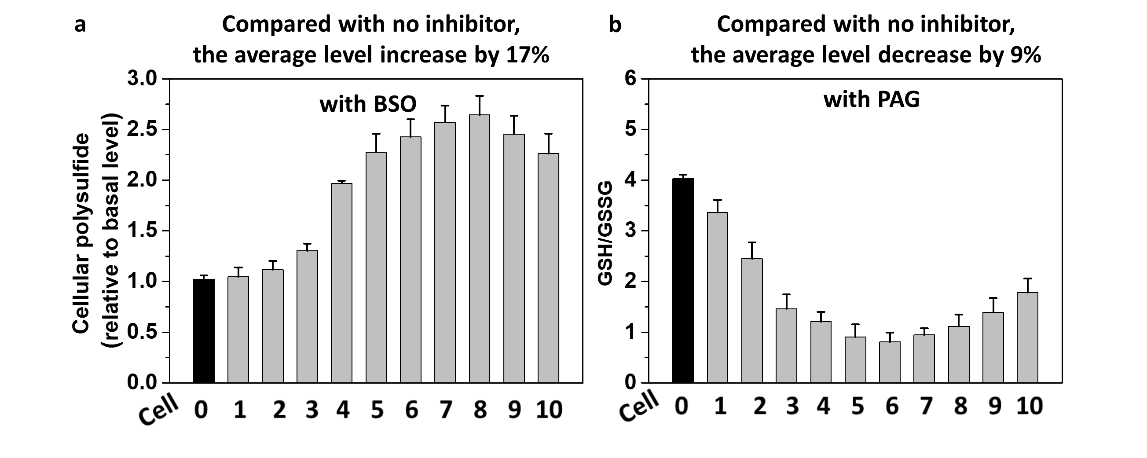


Figure S6. Inhibition of GSH synthesis by BSO slightly enhanced polysulfide synthesis. Conversely, inhibition of polysulfide did not affect GSH levels. (a) Cells were pretreated with BSO for 2 hours, and then treated with GNP for 24 hours to determine cellular polysulfide, measurement of cellular polysulfides were incubated with fluorescent probe Hcy-Mito; (b) Pretreated cells with PAG for 2 hours, then treated with GNP for 24 hours to measure cell GSH/GSSG, total glutathione and reduced glutathione were measured using GSH and GSSG assay kits, respectively.


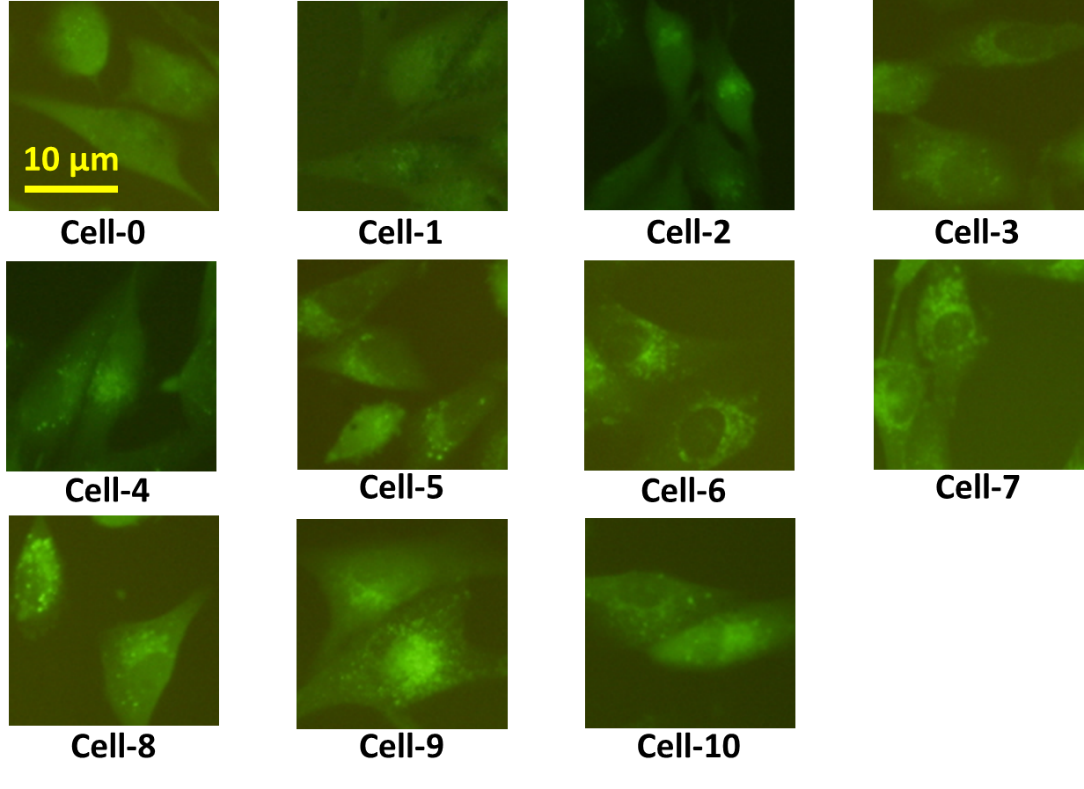


Figure S7. The confocal microscope image of U87 cells is used to reflect autophagy. LC3 autophagy marker protein was introduced into U87 cells by retroviral vector MSCV-IRES-LC3-GFP (2). After treated with GNPs for 24 hours, cells were imaged by inverted fluorescence microscope. LC3 expression corresponds to cellular autophagy levels. Cell autophagy was quantified by counting GFP-LC3 puncta. Image data were analyzed by ImageJ.


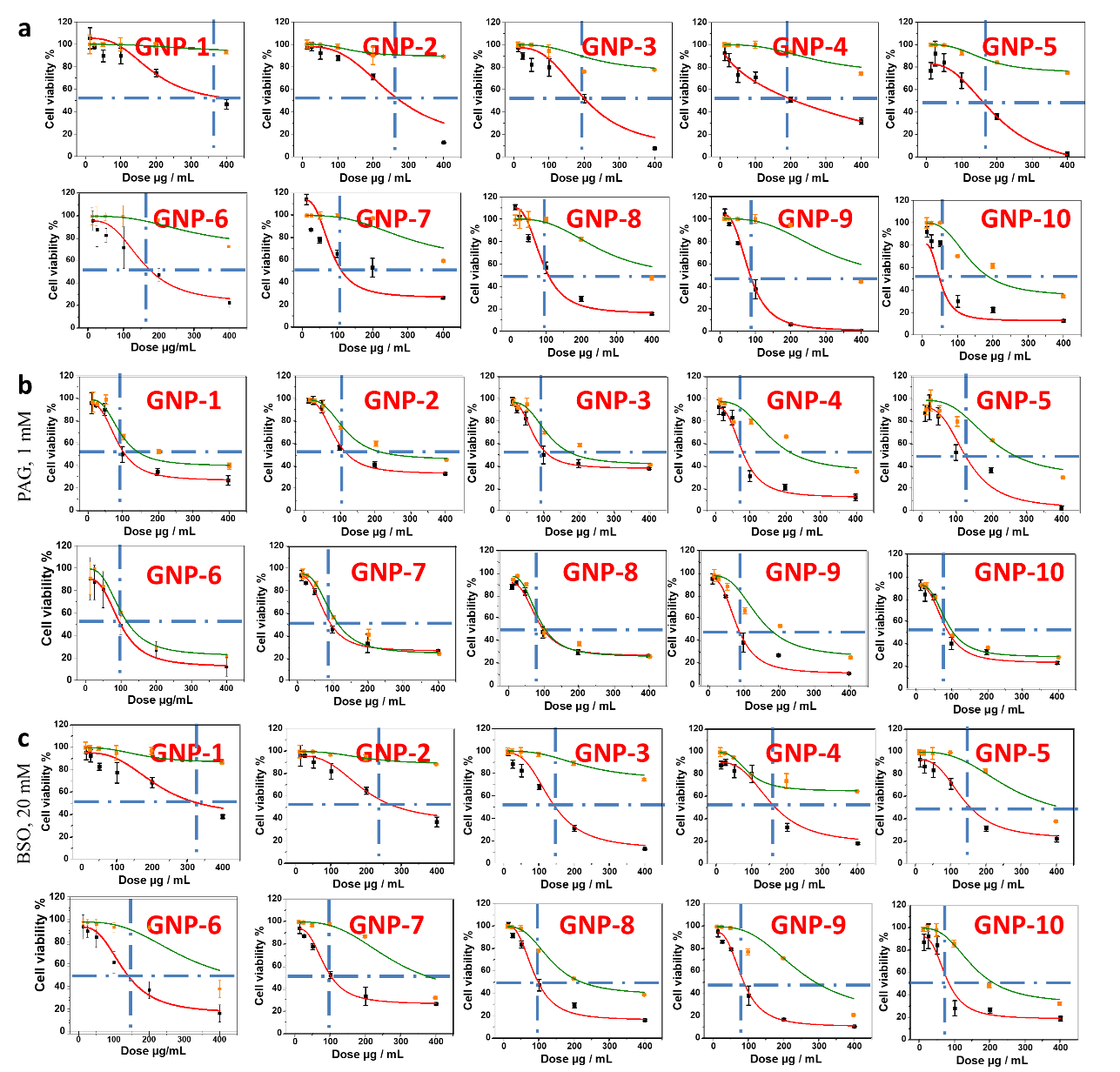


Figure S8. Cytotoxicity of GNPs **1-10**. Cytotoxicity of GNPs **1-10** was measured without inhibitor (a), with CSE inhibitor PAG (1 mM) (b), and ECS inhibitor BSO (20 mM) (c). Viability of cells was measured by CellTiter-Glo® Assay on a VICTORTM X2 Multilabel Plate Reader. The cells were treated with GNPs (0, 12.5, 25, 50, 100, 200, 400 µg/mL) for 48 hours in 96-well plates. After washing with PBS twice, 50 µL of the medium and 50 µL of the CellTiter-Glo® assay working ﬂuid were added in sequence. After gently shaking for 3 min, the plates were incubated for 12 min at room temperature in the dark. Then, 75 µL of the mixture solutions in each well were transferred to another set of white, opaque 96-well plates, and the ﬂuorescence signal intensity was detected by a plate reader (VICTORTM X2, Perkin Elmer, Waltham, MA, USA). Deducting the background signals, cell viability was calculated. Each experiment was done in parallel three times.

Table S1. Summary of differentially expressed mRNA screened.

| Gene name | Log2 (fold change) | | |
| --- | --- | --- | --- |
|  | Cell **3** | Cell **7** | Cell **10** |
| TXNRD2 | -1.815 | -5.76 | 2.85 |
| HSPA1A | -1.625 | -4.16 | -5.2 |
| GSTP1 | -1.545 | -4.44 | -5.47 |
| TXN | -1.545 | 1.55 | 5.99 |
| NFE2L2 | -1.525 | -0.15 | 2.57 |
| SQSTM1 | -1.335 | -3.01 | -2.01 |
| APOE | -1.265 | -1.71 | -3.98 |
| MBL2 | -1.195 | -4.44 | -8.48 |
| GPX4 | -1.095 | 1.91 | 2.44 |
| SIRT2 | -0.995 | -1.41 | -1.38 |
| SRXN1 | -0.955 | -4.38 | -7.15 |
| CDK1 | -0.935 | -5.3 | -2.95 |
| TTN | -0.545 | -4.11 | -7.69 |
| FOXM1 | -0.885 | -3.55 | -5.5 |
| NCF2 | -0.615 | -3.22 | -10.38 |
| PRNP | -0.425 | -2.16 | -6.88 |
| GSTZ1 | -0.265 | -2.12 | -5.23 |
| KRT1 | -0.355 | -1.93 | -10.57 |
| CYBB | -0.135 | -1.51 | -6.23 |
| TPO | -0.765 | -1.37 | -3.41 |
| CCL5 | -0.565 | -1.36 | -6.22 |
| TXNRD1 | -0.355 | -1.15 | 1.91 |
| DUOX1 | -0.495 | -1.15 | 0.1 |
| PXDN | -0.695 | -1.11 | -3.86 |
| MAFK | 0.495 | 1.13 | -1.12 |
| SOD1 | -0.765 | 0.21 | 3.04 |
| ATOX1 | -0.605 | 0.1 | 3.22 |
| UCP2 | -0.565 | 0.01 | 3.75 |
| MGST3 | -0.105 | 0.4 | 3.78 |

Genes with no interactions are not included in the figure for clarity.

References:

1. Huang Y, Yu F, Wang J, Chen L, 2016. Near-infrared fluorescence probe for in situ detection of superoxide anion and hydrogen polysulfides in mitochondrial oxidative stress. *Analytical Chemistry*, 88, 4122-4129.
2. Wu L, Zhang Y, Zhang C, Cui X, Zhai S, Liu Y, Li C, Zhu H, Qu G, Jiang G, Yan B, 2014. Correction to tuning cell autophagy by diversifying carbon nanotube surface chemistry. *ACS Nano*, 8, 5366-5366.
